# Using calculations from the Lives Saved Tool in other global health modelling tools

**DOI:** 10.7189/jogh.15.03012

**Published:** 2025-02-21

**Authors:** Timothy Roberton, Robert McKinnon, Thomas Podkowiak, Jared Schmidt, William Winfrey, Neff Walker

**Affiliations:** 1 Johns Hopkins Bloomberg School of Public Health, Baltimore, Maryland, USA; 2 Avenir Health, Glastonbury, Connecticut, USA

## Abstract

The Lives Saved Tool (LiST) is a widely used software package for modelling changes in child, neonatal, and maternal mortality. Until recently, it has mainly been used as a standalone tool that people manipulate using its desktop or online interface. The developers of LiST have now created a web-based application programming interface (API) allowing other software programmes to interact directly with LiST to use its internal calculations. This opens the door for using LiST within more complex models, for which coverage-to-mortality calculations are only a part, or for building topic-specific tools with a custom interface. The API also allows other software programmes to access the data that has been gathered and maintained by the LiST team on the effectiveness and coverage of 70+ interventions, along with data on mortality rates, cause-of-death structures, and child nutritional status in low- and middle-income countries. In this viewpoint, we describe how we see the API being used and give examples of tools that are already using it. Our hope is that others can now take full advantage of LiST and its 20+ years of development to build their own tools for effective data use in global health.

The Lives Saved Tool (LiST) is widely used in the global health community to estimate the impact of changes in intervention coverage on child, neonatal, and maternal mortality [1]. One of LiST’s strengths is that it comes as a standalone software package (Spectrum) that people can download and run on their computer, with a graphical interface that people can manipulate, clicking to navigate input options and generate results [2]. For many users, this works well. Technical staff in governments, donors, non-governmental organisations, and academic institutions use LiST as part of their work to estimate changes in mortality, entering coverage changes into LiST using the interface and copying results from LiST to use in spreadsheets, narrative text, or presentations [3]. Recently the LiST team built an online version of LiST that allows it to be run in a web browser [4]. This has made LiST more accessible, allowing users to run a model without needing to download or install software, and includes a more streamlined ‘walk through’ interface for creating projections. However, there is another set of use cases for LiST that are not met by either the desktop or online versions of the tool – cases in which other software needs to interact with LiST.

It is often useful to have different software applications talk to each other. Such software-to-software interaction can eliminate manual data manipulation, prevent copy-paste errors, enable automation, and reduce the time needed to perform data analysis. Much of the world’s institutional software operates like this, using what is called an application programming interface (API). An API is a set of commands offered by a software programme that can be used by another software programme to perform tasks, typically by sending data over a local network or via the internet [5]. In recent years, it has become common for web-based platforms to offer an API, to allow more ways for consumers and third-party applications to interact with them. Many companies in the health sector now offer services via an API, whereby external software applications can send a set of input data to the API, the API performs some computation, and the API returns output data back [5]. This type of business model is called ‘Software as a Service’ (SaaS). In general, SaaS improves integration of software across platforms, task automation, versioning control, and innovation of unique applications based on consistent building blocks.

## THE LIST API

For many years, the desktop version of LiST has had an API, allowing other applications to send input data on coverage, baseline mortality, and effectiveness values, and get results without the need for human interaction. However, this API was mainly for internal use by the development team, was not documented or promoted, and was limited to calls from the same local computer on which the LiST desktop software was running. Over the past three years, the software developers of LiST have built a more formal, publicly accessible, web-based API, that enables LiST to be used by other software applications over the internet. This new API allows other software or websites to make a request to the LiST server, send input data for a modelling scenario (country, years, interventions, coverage values, etc.), and get results in return.

**Figure:**
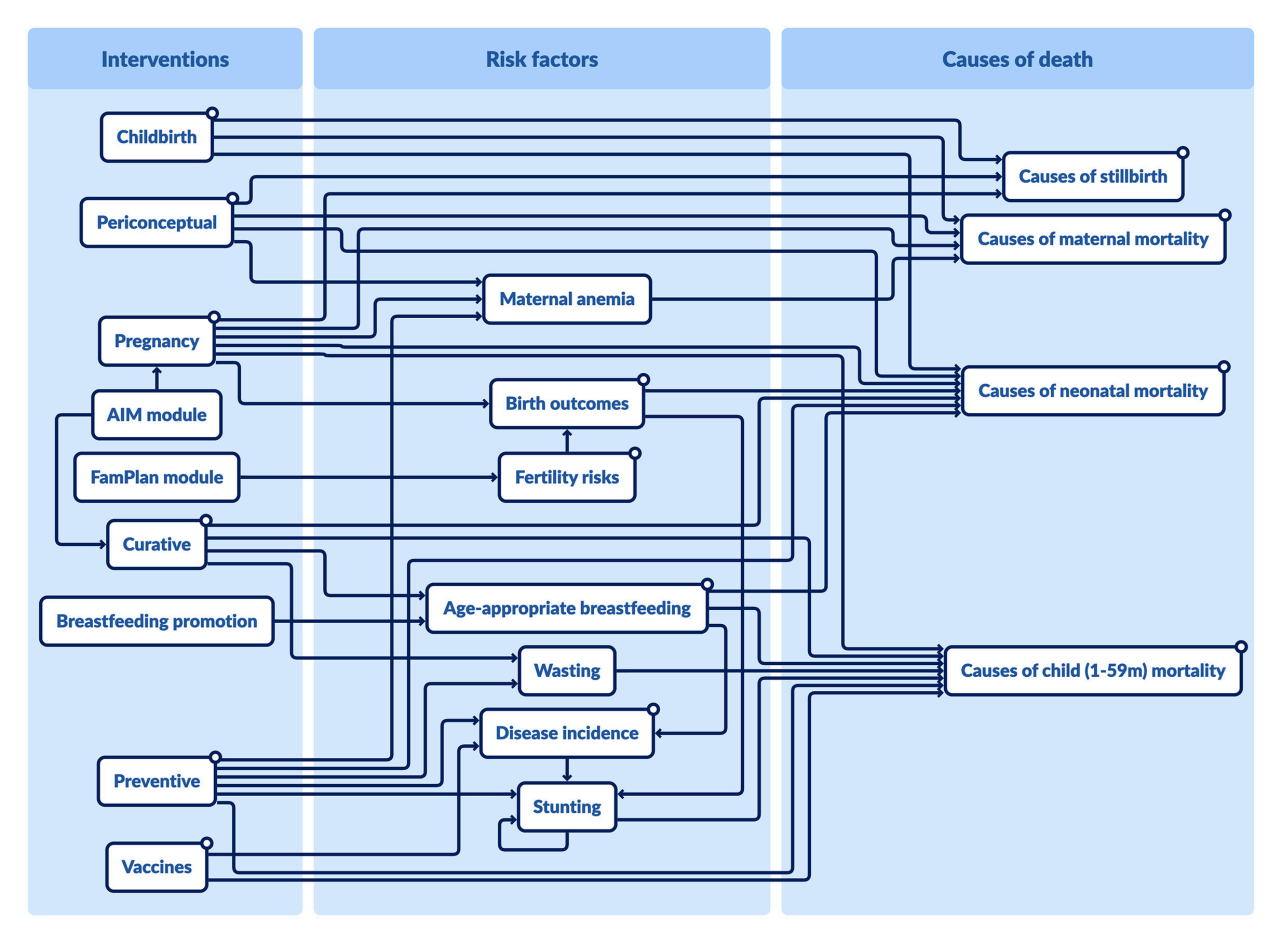
Photo: Obtained from the LiST Visualizer at https://listvisualizer.org. Used with permission.

The LiST API has been set up as a public service for the global health community to use. Until now, users of LiST have predominantly interacted with the desktop or online version of LiST, and likely think of it as a standalone tool. By also serving as a “calculation engine”, we believe LiST can add additional value to the global health community, allowing other modelling tools to be built on top of it. Below we describe some of the ways in which we see the API being used and give examples of tools that are already using it.

## USE CASE 1: LIST CALCULATIONS AS PART OF A LARGER MODEL

The calculations that LiST performs, using coverage changes to estimate mortality changes, are valuable in and of themselves, but there are other modelling scenarios that go beyond the translation of coverage to mortality; for example, more complex modelling efforts for which coverage-to-mortality calculations are only a part, or modelling that puts mortality changes alongside other analytical outputs. The LiST API now allows a more streamlined, programmatic way for LiST to be incorporated into larger modelling processes, without the need for manual transfer of data. For example, in the first year of the COVID-19 pandemic, a team at Johns Hopkins University developed a model that would take routine data from a country HMIS system, run a set of regression analyses to identify observed disruptions to service delivery, and use LiST to estimate the additional deaths due to these disruptions. In this case, the LiST calculations were only one part of a larger model. By using the LiST API, the team was able to automate the entire model, saving time, reducing human error, and increasing replicability.

## USE CASE 2: LIST AS A CALCULATION ENGINE FOR TOPIC-SPECIFIC OR ORGANISATION-SPECIFIC TOOLS

Another possibility that the API allows is for third-party organisations to use LiST as a calculation engine for their own new applications. One reason to do this is to offer users a different interface than the usual LiST interface. Although the LiST interface serves most general purposes, it can be overwhelming for users who only want to adjust a small number of inputs. An organisation may want to create a custom tool that only exposes a specific set of inputs to the user; for example, a nutrition-specific app that only asks the user to consider a handful of nutrition interventions, or an organisation-specific tool that takes a set of select inputs in a particular format. These new tools could present a highly specific interface to the user, but internally use LiST as the calculation engine to perform robust calculations. One example of such a tool is the United Nations Population Fund’s ‘Impact 40’ tool, for strategic planning to address maternal mortality, unmet need for family planning, and female genital mutilation, child marriage, and gender-based violence [6]. The tool takes a set of inputs that are specific to the tool’s subject area, and different from LiST’s usual inputs, yet it uses LiST as part of its internal calculations. Another example is the H-LiST tool, which likewise only uses a subset of LiST’s functionality, for estimating the impact of maternal and child health interventions in humanitarian settings [7].

## USE CASE 3: LIBRARIES FOR STATISTICAL SOFTWARE (R, STATA, SAS, PYTHON)

Many statisticians use general-purpose statistical software, such as R or Stata, rather than domain-specific modelling tools. Statisticians typically write ‘scripts’ for their models or analyses, so that all inputs and calculations are transparently and exactly specified, and so an entire analysis can be reproduced reliably. Another possibility that the LiST API allows is the creation of commands or libraries for R, Stata, and other software, to run mortality-estimation calculations as part of an R script or Stata do-file. Such commands would take inputs from the script or do-file, send them to LiST via an internet request, and return the results to the user, with the details of the request and response handled internally by the command function. We imagine that the creation of these commands or libraries will encourage more statisticians to use LiST, making it possible for them to estimate mortality changes using the statistical software they are already familiar with.

## USE CASE 4: ACCESSING UP-TO-DATE DATA ON COVERAGE, EFFECTIVENESS, AND HEALTH STATUS

In addition to performing calculations, the LiST API can also be used to obtain the data that is stored in LiST, and which is updated regularly by the LiST development team. This includes data on the effectiveness and coverage of 70+ interventions, and the mortality rates, cause-of-death structure, and child nutritional status for all low- and middle-income countries. Many modelling tools may not need to run LiST calculations, but might benefit from accessing this data, using the LiST API to programmatically import coverage and health status data, rather than collecting and storing data separately, or asking users to enter numbers themselves. Such an approach means that as updates are made to the central LiST repository, the custom tool is also immediately kept up to date. One example of a tool that imports data from the LiST API is Optima Nutrition, a model developed by the World Bank to determine funding allocations that minimise stunting, wasting, anaemia, and under-five mortality [8]. Previously, users of Optima Nutrition were required to collect and import their own data, which could be time consuming. Now, when a user wants to import a set of default data, Optima Nutrition sends a request to the LiST API and fetches a set of data that users can browse and use.

We are excited by the future of child and maternal health modelling and encourage organisations and individuals to make the most of the LiST API. The technical details of the API are documented and available for organisations and software developers who want to incorporate LiST as part of a larger model or a new application. The team at Avenir Health are available to support any integration efforts.
